# The effect of digoxin on renal function in patients with heart failure

**DOI:** 10.1186/s12882-021-02562-0

**Published:** 2021-10-26

**Authors:** Parin Shah, Pierpaolo Pellicori, Ian Hanning, Jufen Zhang, Andrew L. Clark, Sunil Bhandari

**Affiliations:** 1 Department of Cardiology, Hull York Medical School, Hull and East Yorkshire Medical Research and Teaching Centre, Castle Hill Hospital, Cottingham, Kingston upon Hull, HU16 5JQ UK; 2 Biochemistry, Pathology department, Hull & East Yorkshire Hospitals NHS Trust, Hull, UK; 3 Clinical Trials Unit, Postgraduate Medical Institute, Faculty of Medical Science, Anglia Ruskin University, Bishop Hall Lane, Chelmsford, Essex, CM1 1SQ UK; 4 Department of Renal Medicine, Hull and East Yorkshire Hospitals NHS Trust and Hull York Medical School, Heslington, UK

**Keywords:** Digoxin, Estimated glomerular filtration rate, Heart failure, Renal function

## Abstract

**Introduction:**

Digoxin is used in patients with chronic heart failure (CHF) who remain symptomatic despite optimal medical treatment. Impaired renal function is commonly associated with CHF. We investigated the relation between digoxin use and change in renal function over time in patients with CHF.

**Methods:**

One thousand two hundred forty-one patients with symptoms and signs of CHF (average age 72 years (64% male), and median NTproBNP 1426 ng/l (interquartile range 632–2897) were divided into four groups: never on digoxin (*N* = 394); digoxin throughout (*N* = 449); started digoxin at some point after baseline (*N* = 367); and stopped digoxin at some point after baseline (*N* = 31). The rate of change of estimated glomerular filtration rate (eGFR) was calculated using linear regression.

**Results:**

Patients on digoxin throughout had a significantly greater rate of decline in eGFR per year than patients not on digoxin throughout (mean (± standard deviation); − 5 (14) ml/min/1.73m^2^ per year *v* − 2 (11) ml/min/1.73m^2^ per year, *P* = 0.02). In those patients who started digoxin during follow up, there was no significant difference in the rate of decline in eGFR before and after starting digoxin. There was no correlation between baseline eGFR (or rate of decline in eGFR) and age, haemoglobin or NTproBNP. Compared to patients taking both angiotensin-converting-enzyme inhibitor (ACEi) or angiotensin receptor blockers (ARB) and beta-blocker (BB), patients who were not taking an ACEi/ARB or BB had a numerically faster rate of decline in eGFR, although this was not statistically significant.

**Conclusion:**

The rate of decline in renal function is greater in patients with CHF who are taking digoxin.

## Introduction

Despite optimal treatment with angiotensin converting enzyme inhibitors(ACEi), beta blockers (BB) and mineralocorticoid receptor antagonists (MRA), some patients with heart failure remain symptomatic. Digoxin is commonly used in patients with atrial fibrillation (AF) as a negative chronotropic agent but is also prescribed in patients with heart failure who are in sinus rhythm for its inotropic and neurohormonal effects [1]. Although digoxin does not improve prognosis, it may improve symptoms [2].

Digoxin has a narrow therapeutic window and therefore needs to be carefully dosed according to age, weight and renal function, and then subsequently monitored [3]. Digoxin clearance varies linearly with estimated glomerular filtration rate (eGFR) and thus any change in renal function affects the efficacy and toxicity of digoxin. Concomitant medications such as amiodarone, quinidine, verapamil, spironolactone or flecainide can also affect the levels of digoxin.

Digoxin inhibits sodium potassium adenosine triphosphatase (Na^+^/K^+^-ATPase) and in the myocardium it increases the intracellular level of sodium and calcium ions in the myocytes thus increasing cardiac contractile force [4]. This mechanism of action of digoxin has also been shown to be an autophagy stimulator [5]. Autophagy, which means ‘self-eating’ in Greek, is an intracellular degradation process for pathogens and damaged organelles by lysosomes [6, 7]. Autophagy maintains cellular homeostasis and energy production, to allow cells to function normally, and is a protective mechanism induced in response to multiple stressors [7, 8]. In the kidneys, autophagy plays an important role in the homeostasis and viability of critical renal cells such as podocytes and tubular epithelial cells [9].

Dysregulation of autophagy is associated with ageing and a variety of pathological conditions, such as neurodegeneration, cardiomyopathy, and cancer.8 Dysregulation of autophagy may be a mechanism which is implicated in the pathogenesis of renal disease, and thus targeting the pathway may be a mechanism for inducing renal protection [9]. In vitro and in vivo studies of renal tissue demonstrate autophagy during hypoxic and ischaemic renal injury [10]. Impaired renal function is commonly associated with chronic heart failure [11]. Stimulating autophagy by digoxin may have renoprotective benefits.

The aim of the present study was to investigate whether there is a significant relationship between digoxin use and change in the decline in renal function over time in patients with chronic heart failure.

## Methods

Consecutive patients referred to a community heart failure clinic, from both primary and secondary care physicians, were enrolled at a single clinic serving a local population of about 500,000 people (The Hull LifeLab). Patients were consented for the use of their medical information as part of the Hull Lifelab registry prior to investigation. PS, PP, JZ and ALC are all co-investigators for the Hull LifeLab registry and had patient level identifiable information as part of the study. Some patients had no prior diagnosis of heart failure and were treatment naive, therefore requiring initiation of guideline-recommended therapy; others had a pre-existing diagnosis of heart failure and had already been initiated on treatment that might, however, require optimisation. Patients with chronic heart failure (defined as symptoms/ signs of heart failure with either reduced left ventricular ejection fraction or amino terminal pro brain type natriuretic peptide; NTproBNP > 220 ng/l) were included.2.

Patients were reviewed by heart failure specialist nurses and doctors at regular intervals, usually at 4 and 12 months, and then annually, unless an appointment was requested sooner by the patient, physician or specialist nurse. Information on demography, symptoms & signs, haematology and biochemistry profiles (including amino terminal pro B type natriuretic peptide (NTproBNP), electrocardiograms and echocardiograms were systematically recorded at each time-point in a dedicated electronic health record stored on a secure NHS server. Titration of treatment was coordinated by the clinic but often implemented by community heart failure nurses or general practitioners.

The patients were divided into four groups on the basis of digoxin use: never on digoxin (*N* = 394); on digoxin throughout (*N* = 449); started digoxin at some point after baseline (*N* = 367); and stopped digoxin at some point after baseline (*N* = 31).

Each patient’s 6 monthly renal function was obtained retrospectively. For the 2 groups with no change in digoxin use, renal function was recorded over 18 months. For the other 2 groups, renal function was obtained 6 and 12 months before starting/stopping of digoxin, at the date of starting/stopping digoxin and 6, 12 and 18 months after starting/stopping digoxin.

The eGFR derived from serum creatinine was calculated using the following equation recommended by National Institute for Health and Care Excellence (NICE) and validated by the local biochemistry laboratory: eGFR ml/min/1.73 m^2^ = 141 × min (S_cr_< /κ, 1)^α^ × max (S_cr_ /κ, 1)^-1.209^ × 0.993^Age^ × 1.018 [if female] × 1.159 [if black] (where **S**_**cr**_ **=** serum creatinine in μmol/L, κ is 61.9 for females and 79.6 for males, α is − 0.329 for females and − 0.411 for males, min indicates the minimum of S_cr_ /κ or 1, and max indicates the maximum of S_cr_/κ or 1) [12].

Change in renal function was calculated as a linear regression using the 6 monthly eGFR from baseline to 12 months. At least 3 values were used to produce a linear regression line. For the patients with heart failure who started/ stopped digoxin after baseline, the rate of change in renal function before and after the change in digoxin use was calculated.

The research conforms to the Helsinki declaration and ethics approval was given by the Hull and East Riding Local Research Ethics Committee for the use of the patient records. Informed consent was obtained from all subjects or if subjects are under 18, from a parent and/or legal guardian.at the point of first diagnosis of heart failure to use their medical record for research purposes.

### Statistical analysis

Categorical data are presented as number (percentage); normally distributed continuous data as mean (standard deviation), non-normally distributed continuous variables as median (interquartile range). One-way ANOVA was used to compare means of more than two groups for continuous variables and chi-square tests were used for categorical variables.

For each patient, the linear regression of eGFR against time was calculated, both before and after starting digoxin (for the relevant groups). Independent t tests were used to compare the means between two groups and paired t tests were used to compare the means within a group.

Statistical analyses were performed with SPSS (version 22) and STATA (version 14.1, Stata-Corp) statistical software. All tests are two-sided, with a *p*-value of< 0.05 considered significant.

## Results

Baseline characteristics are shown in Table 1. There was a total of 1241 patients, with a mean age 72 (12) years; 64% of the patients were male. The majority were in New York Heart Association class II or III, with a median NT pro BNP of 1426 (632–2897) ng/l. There was no difference between the groups in NTproBNP, haemoglobin and renal function. At baseline, 75% of patients were taking an ACEi or ARB, 58% a beta-blocker and 25% an MRA.

**Table 1:** baseline characteristics of patients with heart failure, divided into 4 groups. BMI: body mass index, NYHA class: New York Heart Association class, LV: Left Ventricular, NTproBNP: N-terminal pro b-type natriuretic peptide, eGFR: estimated glomerular filtration rate, ACEi: angiotensin-converting-enzyme inhibitor, ARB: Angiotensin II receptor blockers, MRA: mineralocorticoid receptor antagonists, IHD: ischaemic heart disease, COPD: chronic pulmonary obstructive disease. * *P* value between patients taking digoxin and patients not taking digoxin at baseline. ** P value between 4 groups of patients (digoxin throughout, never on digoxin, started digoxin after baseline, stopped digoxin after baseline)

	Missing values	Digoxin at baseline		* ***P*** value	Digoxin use throughout follow up				** ***P*** value
		Yes	No		Digoxin through out	Never on digoxin	Started Digoxin after baseline	Stopped Digoxin after baseline	
		(***N*** = 480)	(***N*** = 761)		(***N*** = 449)	(***N*** = 394)	(***N*** = 367)	(***N*** = 31)	
**Age (years)**	0	73 (11)	71 (11)	0.07	73 (11)	71 (11)	72 (10)	72 (11)	0.04
**Sex (% Male)**	0	291 (61)	505 (66)	0.04	267 (59)	261 (66)	244 (66)	24 (77)	0.07
**BMI (kg/m**^**2**^**)**	10	28 (6)	29 (6)	< 0.001	28 (6)	29 (6)	29 (7)	28 (6)	0.95
**Sinus rhythm (%)**	0	91 (19)	505 (66)	< 0.001	76 (17)	294 (75)	211 (58)	15 (48)	< 0.001
**NYHA class**									
**I (%)**	21	72 (15)	149 (20)	0.34	66 (15)	101 (26)	48 (13)	6 (19)	< 0.001
**II (%)**		230 (48)	326 (43)		212 (47)	172 (44)	154 (42)	18 (58)	
**III (%)**		160 (33)	249 (33)		154 (34)	107 (27)	142 (39)	6 (19)	
**IV (%)**		12 (3)	22 (3)		12 (3)	7 (2)	15 (4)	0 (0)	
**LV impairment**									
**Normal – trivial (%)**	32	169 (35)	226 (30)	0.01	162 (36)	165 (42)	61 (17)	7 (23)	
**Mild – mild to moderate (%)**		115 (24)	185 (24)		108 (24)	90 (23)	95 (26)	7 (23)	< 0.001
**Moderate - Moderate to severe (%)**		176 (37)	338 (44)		161 (36)	129 (33)	209 (57)	15 (48)	
**Bloods**									
**NTproBNP (ng/L)**	252	1891 (972–3506)	1081 (440–2469)	0.02	1914 (1003–3515)	802 (302–1835)	1513 (771–3312)	1335 (630–4279)	0.98
**eGFR (ml/min/1.73m**^**2**^**)**	19	60 (21)	55 (21)	< 0.001	61 (21)	58 (21)	51 (21)	52 (19)	0.33
**Urea (mmol/L)**	10	8.0 (4.7)	8.5 (5.1)	0.07	8 (5)	8 (5)	14 (2)	9 (5)	0.07
**Haemoglobin (g/dL)**	133	13.5 (1.9)	13.4 (1.7)	0.80	13 (2)	13 (2)	14 (2)	13 (2)	0.11
**Albumin (g/L)**	94	37 (4)	38 (4)	< 0.001	37 (4)	38 (3)	38 (4)	37 (4)	< 0.001
**Medications**									
**ACEi (%)**	0	315 (66)	502 (66)	0.90	290 (65)	249 (63)	253 (69)	25 (81)	0.11
**ARB (%)**	0	45 (9)	72 (10)	0.96	44 (10)	41 (10)	31 (8)	1 (3)	0.58
**Beta blockers (%)**	0	254 (53)	460 (60)	0.01	238 (53)	242 (61)	218 (59)	16 (52)	0.07
**MRA (%)**	0	144 (30)	160 (21)	< 0.001	136 (30)	68 (17)	92 (25)	8 (26)	< 0.001
**Diuretic (%)**	0	404 (84)	517 (68)	< 0.001	376 (84)	231 (59)	286 (78)	28 (90)	< 0.001
**Co morbidities**									
**Diabetic (%)**	0	108 (23)	184 (24)	0.50	102 (23)	104 (26)	80 (22)	6 (19)	0.41
**Hypertension (%)**	0	151 (32)	285 (38)	0.03	140 (31)	163 (41)	122 (33)	11 (36)	0.01
**IHD (%)**	0	164 (34)	416 (55)	< 0.001	149 (33)	205 (52)	211 (58)	15 (48)	0.00
**COPD (%)**	0	61 (13)	70 (9)	0.05	57 (13)	30 (8)	40 (11)	4 (13)	0.12

**Fig. 1:**
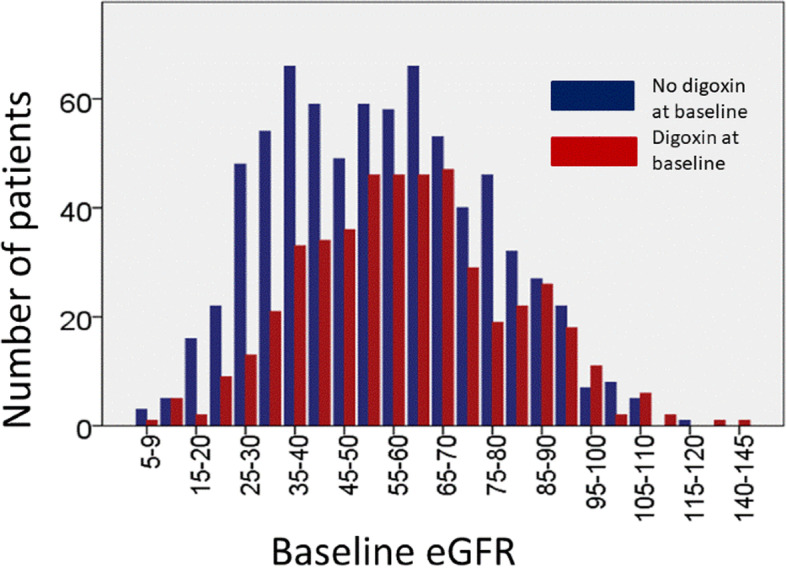
Distribution of renal function at baseline in patients on digoxin or not on digoxin at baseline

Baseline eGFR was slightly lower in patients not on digoxin at baseline than in those taking digoxin (Table 1, Fig. 1). Compared to patients not on digoxin at baseline, patients taking digoxin at baseline were less likely to be in sinus rhythm, had a higher NTproBNP and were more likely to be taking a diuretic and/or a MRA. (Table 1).

In the overall population, eGFR declined at a rate of − 3.2 (11.3) ml/min/1.73m^2^ per year. Compared to patients never on digoxin, patients on digoxin throughout had a significantly higher rate of decline in eGFR per year. In patients who started digoxin after baseline, the rate of decline in eGFR was numerically greater after starting digoxin but the change was not statistically significant. (Table 2).

**Table 2:** rate of decline in eGFR according to the use of digoxin in patients with heart failure. eGFR: estimated glomerular filtration rate

	Group 1	Group 2	Group 3
	Never on digoxin	Digoxin throughout	Started Digoxin after baseline
Rate of decline of eGFR (ml/min/1.73m^2^per year)	−2 (10)	−5 (13)	Off digoxin −1 (9)
			After digoxin −4 (23)
	P = 0.02		*P* = 0.31

There was a correlation between baseline eGFR and body mass index. There was no correlation between baseline eGFR (or rate of decline in eGFR) and age, haemoglobin or NTproBNP. (Table 3) Patients with worse symptoms (NYHA class III/IV), who were taking loop diuretics or who had diabetes, hypertension or ischaemic heart disease had a significantly lower baseline eGFR but no significant difference in the rate of decline in eGFR. (Table 4) Compared to patients taking both ACEi/ARB and BB, patients who were not taking an ACEi/ARB or BB had a numerically faster rate of decline in eGFR, although this was not statistically significant. (Tables 4 and 5).

**Table 3:** correlation of baseline eGFR and rate of decline in eGFR with continuous variables (demographic and blood variables). eGFR: estimated glomerular filtration rate, BMI: body mass index, NTproBNP: amino terminal pro brain natriuretic peptide

Variable	Baseline eGFR (ml/min/1.73m^**2**^)			Rate of decline in eGFR (ml/min/1.73m^**2**^per year)		
	Missing value	Pearson Correlation Coefficient	***P*** value	Missing value	Pearson Correlation Coefficient	***P*** value
**Age**	18	−0.48	< 0.01	465	0.01	0.89
**Weight**	26	0.14	< 0.01	468	0.09	0.02
**BMI**	29	0.70	0.01	469	0.05	0.15
**Sodium**	23	−0.04	0.14	470	0.07	0.04
**Urea**	22	−0.59	< 0.01	470	0.18	< 0.01
**Haemoglobin**	146	0.31	< 0.01	540	0.05	0.21
**NTproBNP**	262	−0.28	< 0.01	613	−0.03	0.48

**Table 4:** correlation of baseline eGFR and rate of decline in eGFR with categorical variables. eGFR: estimated glomerular filtration rate, NYHA: New York Heart Association, LV: left ventricular, ACEi: angiotensin-converting-enzyme inhibitor, ARB: angiotensin receptor blockers, BB: beta blocker, MRA: mineralocorticoid receptor antagonist, IHD: ischaemic heart disease

Variable	Missing values	Mean Baseline eGFR (ml/min/1.73m^**2**^)		***P*** value	Missing values	Mean Rate of decline in eGFR (ml/min/1.73m^**2**^ per year)		***P*** value
**Sex: male** ***v*** **female**	18	58 (22)	54 (19)	< 0.01	465	− 2.5 (11.0)	−4.3 (11.8)	0.04
**Sinus rhythm: yes** ***v*** **no**	30	57 (22)	57 (20)	0.63	471	−2.9 (11.0)	−3.4 (11.6)	0.61
**NYHA class: III/IV v I/II**	41	54 (21)	59 (21)	< 0.01	471	−2.6 (10.6)	−4.2 (12.4)	0.06
**LV dysfunction: moderate/severe** ***v*** **normal/mild**	51	56 (22)	57 (20)	0.59	483	−3.0 (11.2)	− 3.1 (11.3)	0.88
**ACEi /ARB: yes** ***v*** **no**	19	57 (21)	56 (22)	0.22	465	−2.4 (10.8)	−5.7 (12.8)	0.04
**BB: yes** ***v*** **no**	19	58 (22)	56 (20)	0.18	465	−2.2 (10.9)	−4.6 (11.8)	< 0.01
**MRA: yes** ***v*** **no**	19	58 (23)	57 (20)	0.30	465	−3.4 (13.6)	−3.0 (10.3)	0.66
**Loop diuretic: yes** ***v*** **no**	19	54 (21)	64 (19)	< 0.01	465	−3.3 (11.7)	−2.6 (10.1)	0.44
**Diabetes: yes** ***v*** **no**	19	54 (21)	58 (21)	< 0.01	465	−3.0 (10.5)	−3.2 (11.6)	0.78
**Hypertension: yes** ***v*** **no**	19	55 (20)	58 (22)	< 0.01	465	−2.8 (10.4)	−3.3 (11.8)	0.51
**IHD: yes** ***v*** **no**	19	53 (21)	60 (21)	< 0.01	465	−2.3 (10.9)	−3.9 (11.6)	0.05

**Table 5:** Baseline eGFR and rate of change in eGFR according to the combination of ACEi/ARB and BB. eGFR: estimated glomerular filtration rate, ACEi: angiotensin-converting-enzyme inhibitor, ARB: angiotensin receptor blockers, BB: beta blocker

Medications	missing	Baseline eGFR (ml/min/1.73m^**2**^)	***P*** value	missing	Rate of change of eGFR (ml/min/1.73m^**2**^ per year)	***P*** value
**No ACEi/ARB and no BB**	5	57 (21)	0.42	81	−6.6 (13.9)	0.60
**ACEi/ARB, No BB**	6	56 (19)		142	−3.6 (10.4)	
**BB, No ACEi/ARB**	1	54 (22)		59	−4.3 (11.1)	
**ACEi/ARB and BB**	7	58 (22)		183	−1.9 (10.9)	

Compared to patients in sinus rhythm, those in atrial fibrillation (AF) were older, had a significantly higher NTproBNP and were more likely to be on a diuretic. There was no difference in baseline eGFR (or rate of decline in eGFR) between patients in sinus rhythm and AF. (Table 6).

**Table 6:** Difference in baseline characteristics and rate of change in eGFR, in patients with sinus rhythm and AF. AF: atrial fibrillation, BMI: body mass index, NYHA class: New York Heart Association class, LVSD: Left ventricular, NTproBNP: N-terminal pro b-type natriuretic peptide, eGFR: estimated glomerular filtration rate, ACEi: angiotensin-converting-enzyme inhibitor, ARB: Angiotensin II receptor blockers, MRA: mineralocorticoid receptor antagonists, IHD: ischaemic heart disease, COPD: chronic pulmonary obstructive disease

	Missing values	Heart rhythm		*P value
		Sinus	AF	
		(***N*** = 605)	(***N*** = 636)	
**Age (years)**	0	70 (11)	74 (10)	< 0.001
**Sex (% Male)**	0	393 (65)	403 (63)	0.56
**BMI (kg/m**^**2**^**)**	10	29 (6)	29 (6)	0.63
**NYHA class**				
**I (%)**	21	132 (22)	89 (14)	< 0.001
**II (%)**		269 (45)	287 (45)	
**III (%)**		176 (29)	233 (37)	
**IV (%)**		17 (3)	17 (3)	
**LV impairment**				
**Normal – trivial (%)**	32	172 (28)	223 (35)	< 0.001
**Mild – mild to moderate (%)**		129 (21)	171 (27)	
**Moderate - Moderate to severe (%)**		291 (48)	223 (35)	
**Bloods**				
**NTproBNP (ng/L)**	252	842 (313–2260)	1920 (1042–3383)	< 0.01
**eGFR (ml/min/1.73m**^**2**^**)**	18	57 (22)	57 (20)	0.64
**Rate of decline in eGFR (ml/min/1.73m**^**2**^ **per year)**	471	−2.9 (11.0)	−3.4 (11.6)	0.61
**Urea (mmol/L)**	10	8.1 (4.8)	8.5 (5.2)	0.12
**Haemoglobin (g/dL)**	133	13.4 (1.7)	13.6 (1.9)	0.11
**Albumin (g/L)**	95	38 (40	37 (4)	0.08
**Medications**				
**ACEi (%)**	0	405 (67)	412 (65)	0.42
**ARB (%)**	0	56 (9)	61 (10)	0.84
**Beta blockers (%)**	0	344 (57)	370 (58)	0.64
**MRA (%)**	0	144 (24)	160 (25)	0.58
**Diuretic (%)**	0	416 (69)	505 (79)	< 0.001
**Co morbidities**				
**Diabetic (%)**	0	149 (25)	143 (23)	0.37
**Hypertension (%)**	0	215 (36)	221 (35)	0.77
**IHD (%)**	0	331 (55)	249 (39)	< 0.001
**COPD (%)**	0	69 (11)	62 (10)	0.34

## Discussion

Our findings do not support the hypothesis that digoxin might have a protective effect against progressive renal dysfunction in patients with heart failure. We found that renal function declined with time in the whole cohort but that the rate of decline was significantly faster in patients who were already taking digoxin at baseline. Renal function declines with age [13, 14], and more rapidly in patients with heart failure [11]. Treatment of heart failure with ACEi and MRAs almost inevitably lead to a degree renal dysfunction, in part due to haemodynamic changes [11, 15].

Digoxin has been used in patients with heart failure for over 200 years [16], but there is no clear evidence of a benefit on mortality. In the DIG trial, conducted in patients with heart failure due to left ventricular systolic dysfunction who were in sinus rhythm, digoxin reduced hospitalizations due to heart failure by 7.9% but had no significant effect on all-cause mortality.3 In a subgroup analysis, patients with more severe symptoms and signs (NYHA class III/IV or left ventricular ejection fraction less than 25% or who had a cardiothoracic ratio more than 55%) digoxin use was associated with a reduction in 2 year heart failure mortality and hospitalization.3 In contrast, meta-analyses by Vamos and colleagues and Bavishi and colleagues suggested that digoxin was associated with an increase in mortality both in patients with HF and in patients with AF by 21 and 15%, respectively: however, these meta-analyses were conducted on observational and registry studies [3, 17]. Digoxin had no effect on mortality in a meta-analysis of 7 randomised control trials with a control arm in both patients with HF and those with AF [18]. As a consequence of the lack in mortality benefit, digoxin tends to be reserved particularly for those who remain symptomatic despite first line treatment with ACEi, BB and MRA.2.

The effect of worsening renal function on digoxin toxicity is well known [19]. In end-stage renal disease, the fluctuating concentration of potassium during dialysis may increase the risk of digoxin toxicity. In patients with end stage renal failure, mortality increases with increasing serum digoxin levels [20]. The sodium/potassium ATPase pump normally causes sodium to leave cells and potassium to enter cells, blocking this mechanism using digoxin results in higher serum potassium levels. In a study by Edner and colleagues of 10 healthy subjects, compared to no digitaliation, patients given 0.37–0.50 mg of digoxin daily for 10 days had an increase in serum potassium by 0.19 ± 0.23 mmol/l (*p* < 0.05) [21] When renal potassium excretion is reduced due to reduction in kidney function, even slow-acting glyeosides should provoke hyperkalemia [22].

Whether digoxin affects renal function is not fully understood. We found that patients on digoxin had a significantly faster rate of decline in eGFR. However, in subgroup analysis of patients in the DIG trial who had their creatinine measured at 1 year, renal function improved more in patients in the digoxin group than in those taking placebo [23]. Mortality in DIG did not vary with the use of digoxin in relation to renal function, although lower doses of digoxin were prescribed to patients with the lowest estimated glomerular filtration rate (eGFR), presumably as a result of dose adjustment for renal function [24].

Digoxin is a negatively chronotropic and positively inotropic agent. It inhibits the Na^+^/K^+^-ATPase resulting in an increase in intracellular sodium and calcium ions leading to its positive inotropic effect. The negatively chronotropic effect is via an incompletely understood vagotonic effect. Apart from its cardiac uses, cardiac glycosides have been implicated in the regulation of many other physiological and pathophysiological processes. The cardiac glycoside, neriifolin, reduces cerebral infarct size in rodent cerebral hypoxia–ischemia models [25]. Digitalis blocks cell proliferation and non-toxic doses of digitalis can induce apoptosis in different malignant cell lines [26]. Digoxin might thus be useful in diseases associated with autotic cell death or autophagy in the kidneys [27].

Cardiotonic steroids are endogenous ligands of the Na^+^/K^+^-ATPase and are implicated in the regulation of natriuresis and vascular tone [28–30]. There is an increased circulating level of the cardiotonic steroid, ouabain, in patients with severely impaired left ventricular function [31], which has been shown to predict progression of heart failure both in patients with idiopathic dilated cardiomyopathy and in those with left ventricular hypertrophy in end stage renal failure [32, 33]. In partially nephrectomized rats, diastolic dysfunction and cardiac fibrosis are accompanied by raised levels of cardio tonic steroids. Cardiotonic steroids such as ouabain, digoxin, marinobufagenin and telocinobufagin have all been found to be raised in the plasma of experimental animals and patients with CKD [34]. A four week infusion of the cardiotonic steroid, marinobufagenin induce renal fibrosis in rats [35]. Serum from patients with chronic renal failure and diastolic dysfunction caused inhibition of Na,K-ATPase purified from dog kidney and impaired recovery of cardiac myocyte calcium concentration as well as impaired relaxation of myocytes isolated from Sprague-Dawley rats [36].

The use of digoxin in patients with heart failure (where serum levels of digoxin are > 1.2 ng/ml) is associated with an 11.8% increase in mortality[37]. However, Komiyama and colleagues reported elevated plasma levels of cardiotonic steroids in patients with end stage renal failure, far in excess of 1.2 ng/ [38]. Cardiotonic steroids at the concentrations detected in patients and animals with CKD can potentially inhibit cardiac Na+/K + -ATPase enzyme activity. In partially nephrectomized rats, active immunization against the cardiotonic steroid, marinobufagenin, causes a dramatic reduction in cardiac hypertrophy and fibrosis. Adding digoxin to endogenous cardiotonic steroids had a synergistic effect on the inhibition of Na+/K + -ATPase [34].

The association between worse renal function and digoxin use may be related to atrial fibrillation. We found a very strong association between atrial fibrillation and digoxin usage. The development of AF is associated with a two fold increase in risk of developing end stage renal disease in patients with chronic kidney disease (CKD), independent of baseline eGFR [39]. Animal data show that AF can cause renal vasoconstriction and decreased renal blood flow and even renal fibrosis or possibly renal micro-infarcts [40, 41]. In 386 patients with AF treated by ablation, patients who were arrhythmia free during the first year had an increase in eGFR whilst those who had recurrence of their arrhythmia had a reduction in eGFR (3 ± 8 ml/min/1.73m^2^ vs − 2 ± 8 ml/min/1.73m^2^, respectively: *P*< 0.0001) [42, 43].

An additional reason for the greater decline in renal function associated with the use of digoxin may be, at least in part, because digoxin is more likely to be used in patients with more severe heart failure (atrial fibrillation, higher NTproBNP and those already on diuretics) despite treatment with ACEi, beta blockers and MRA.2.

## Limitations

This was a retrospective analysis of patients with heart failure and therefore there may be confounding factors contributing to the changes in renal function.

Patients may have other co-morbidities such as diabetes and hypertension which may contribute to impaired renal function. We did not categorise patients into types of renal disease.

We have not reported the level of proteinuria as this is a surrogate marker and not a hard outcome measure. It may help separate patients into sub-groups but we did not want to do this due to the possible introduction of error.

## Conclusions

The rate of decline in renal function is greater in patients with CHF who are taking digoxin.

## Data Availability

The datasets used and/or analysed during the current study available from the corresponding author on reasonable request.
